# Disclosing Early Excited State Relaxation Events in Prototypical Linear Carbon Chains

**DOI:** 10.1021/jacs.3c04163

**Published:** 2023-08-01

**Authors:** Piotr Kabaciński, Pietro Marabotti, Daniele Fazzi, Vasilis Petropoulos, Andrea Iudica, Patrick Serafini, Giulio Cerullo, Carlo S. Casari, Margherita Zavelani-Rossi

**Affiliations:** † Dipartimento di Fisica, Politecnico di Milano, piazza Leonardo da Vinci 32, 20133 Milano, Italy; ‡ Dipartimento di Energia, Politecnico di Milano, via G. Ponzio 34/3, 20133 Milano, Italy; § Dipartimento di Chimica “Giacomo Ciamician”, Università degli studi di Bologna, via F. Selmi 2, 40126 Bologna, Italy; ∥ Istituto di Fotonica e Nanotecnologie IFN-CNR, piazza Leonardo da Vinci 32, 20133 Milano, Italy

## Abstract

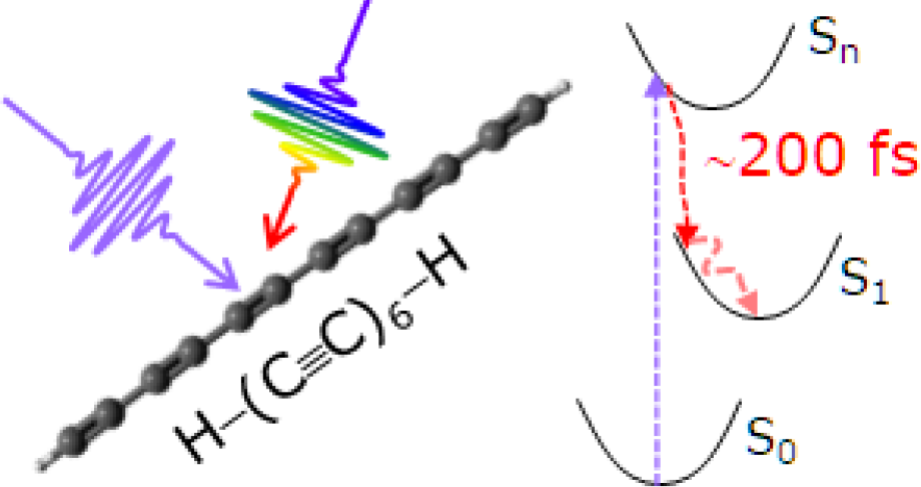

One-dimensional (1D) linear nanostructures comprising *sp*-hybridized carbon atoms, as derivatives of the prototypical allotrope known as carbyne, are predicted to possess outstanding mechanical, thermal, and electronic properties. Despite recent advances in their synthesis, their chemical and physical properties are still poorly understood. Here, we investigate the photophysics of a prototypical polyyne (i.e., 1D chain with alternating single and triple carbon bonds) as the simplest model of finite carbon wire and as a prototype of *sp*-carbon-based chains. We perform transient absorption experiments with high temporal resolution (<30 fs) on monodispersed hydrogen-capped hexayne H—(C≡C)_6_—H synthesized by laser ablation in liquid. With the support of computational studies based on ground state density functional theory (DFT) and excited state time-dependent (TD)-DFT calculations, we provide a comprehensive description of the excited state relaxation processes at early times following photoexcitation. We show that the internal conversion from a bright high-energy singlet excited state to a low-lying singlet dark state is ultrafast and takes place with a 200 fs time constant, followed by thermalization on the picosecond time scale and decay of the low-energy singlet state with hundreds of picoseconds time constant. We also show that the time scale of these processes does not depend on the end groups capping the *sp*-carbon chain. The understanding of the primary photoinduced events in polyynes is of key importance both for fundamental knowledge and for potential optoelectronic and light-harvesting applications of low-dimensional nanostructured carbon-based materials.

## Introduction

Linear nanostructures comprising *sp*-hybridized carbon atoms are one-dimensional (1D) materials (i.e., one-atom-thick chains) featuring peculiar structure–property relationships as a result of extended π-electron conjugation and strong electron–phonon coupling.^1^ The ideal infinite system (i.e., carbyne) represents the lacking carbon allotrope beside graphite and diamond and is predicted to possess outstanding mechanical, thermal, and electronic properties.^1−5^ The *sp*-hybridization allows to maximize the π-electron conjugation, leading to two possible structural and electronic configurations: (i) the equalized bond structure (i.e., the cumulenic form =(C=C)_*n*_=, with a sequence of double bonds) as an ideal 1D metal showing a zero band gap; (ii) the alternated bond structure (i.e., the polyynic form —(C≡C)_*n*_—, with a sequence of alternating single and triple bonds),^6^ which is favored by Peierls’ instability and features a finite band gap.

While carbyne has been the focus of many theoretical studies, its experimental realization through chemical or physical processes is still in its infancy. The closest available system to the ideal carbyne is a long linear carbon chain encapsulated in the core of a double-walled carbon nanotube, the so-called confined carbyne.^7^ This system has been shown to feature the strongest resonance Raman cross section ever reported, with a very large Huang–Rhys factor indicating a strong electron–phonon coupling.^8,9^ However, the electronic and optical characteristics of confined carbyne, such as the band gap and the vibrational frequencies of the Raman-active mode, depend on the type of encapsulating system, making it difficult to assess the intrinsic carbyne properties by looking for the saturation of size-dependent effects.^11−16^ Size (chain length) and termination (end-capping groups) dependent effects are indeed dominating in finite-size *sp*-carbon chains or carbon atomic wires, making such systems attractive for developing materials with tailored properties.^17−19^ For example, a field-effect transistor with promising field-effect charge mobility has been recently realized using short cumulenic wires (i.e., three C=C bonds) as active material.^20,21^

The strong relationship between structural, electronic, and vibrational properties in linear carbon chains has been exploited for their characterization mainly through UV–vis absorption and Raman spectroscopy. Recently, resonant Raman in the UV in combination with UV–vis absorption has been used to retrieve the electronic and vibrational structure of the ground and first excited states of short polyynic chains, showing a peculiar size-dependent electron–phonon coupling.^22^ This was possible because of the neat vibronic effects characterizing the absorption spectrum. Steady-state Raman and UV–vis absorption spectroscopy, however, do not provide any information about the properties of the excited states.

To date, only a few studies report on the excited state dynamics of carbynes.^12,23,24^ Due to the difficulties in synthesizing simple and stable systems and performing high temporal resolution ultrafast spectroscopy in the challenging UV spectral range, only partial information on specific systems could be obtained. Fazzi et al.^23^ studied dinaphthyl end-capped polyynes revealing, by visible ultrafast transient absorption (TA) spectroscopy, the intersystem crossing (ISC) process which populates a triplet state from an excited singlet state, with 30 ps formation time. Movsisyan et al.^24^ used UV-NIR-IR TA spectroscopy to study the excited-state dynamics of an hexayne chain stabilized with aryl-based end groups (i.e., tris(3,5-di-*tert*-butylphenyl)methyl), either free or threaded through a phenanthroline–rotaxane macrocycle. The system showed the formation of a dark S_1_ excited state via an internal conversion (IC) process from higher-lying singlet states (S_*n*_), followed by a slow ISC to the triplet state. IC was characterized by a few-picoseconds time constant (∼1 ps for the free hexayne, 2–3 ps for the rotaxane-encapsulated chain) and ISC by ∼0.4 ns time constant. Very recently,^12^ a study on oligoynes with different terminal groups and chain lengths (i.e., number of triple bonds *n* = 4–12) showed, through ultrafast TA spectroscopy, short-lived S_*n*_ excited states decaying to S_1_ with time constants between 1.4 and 8.8 ps (depending on *n* and the end group), followed by ISC toward the triplet state on longer time scales (0.1–4.7 ns).

Overall, the investigated systems have bulky sterically hindered end groups (e.g., aryl-based)^25^ that are needed to stabilize the *sp*-chain, however possibly perturbing the overall excited state energies and potential energy profiles when compared with the prototypical (ideal) hydrogen-capped system H—(C≡C)_*n*_—H. The questions about the intrinsic excited state properties of the *sp*-carbon backbone and the influence of end groups on the relaxation dynamics of the carbon wires are still open. Moreover, despite the expected ultrafast dynamics due to the extended π-electron conjugation, the early events characterizing the fate of the photoexcited singlet state and the IC process occurring on the 100 fs time scale have not been addressed yet. In this context, hydrogen-capped polyynic *sp*-carbon chains represent a prototypical case of finite-size carbyne with the simplest possible termination and can be exploited as a model system to investigate the intrinsic photophysical properties of short *sp*-carbon chains.

It has been proposed that the excited state dynamics of polyynes bears similarity to that of carotenoids, which are conjugated *sp*^2^-carbon chains consisting of alternating single and double bonds. Carotenoids are found in all photosynthetic light-harvesting complexes and play many key roles, including harvesting the blue-green components of the solar spectrum, photoprotection by quenching singlet oxygen, and regulation of the photosynthetic activity through the so-called nonphotochemical quenching mechanism.^26^ Carotenoids display a peculiar photophysics, whereby the lowest energy excited state S_1_ has the same symmetry (e.g., ^2^A_g_) as the ground state (e.g., ^1^A_g_) and is therefore optically dark.^27^ Generally, photoexcitation reaches the higher-lying dipole-allowed excited state S_*n*_ (the B_1u_ state), from which an ultrafast IC to the dark state S_1_ occurs on the ∼100 fs time scale, possibly mediated by intermediate states.^27−29^ Despite the expected similarity, the complete photoexcitation scenario has not yet been analyzed and understood for *sp*-carbon systems such as polyynes and, in particular, for the simple hydrogen-capped (unsubstituted) carbon chain.

Here we perform UV ultrafast TA spectroscopy with <30 fs time resolution and broad spectral coverage on the hydrogen-capped polyyne H—(C≡C)_6_—H, as the simplest possible model of finite *sp*-carbon wire. Our experiments are combined with a detailed computational study based on ground state density functional theory (DFT) and excited state time-dependent (TD)-DFT calculations. We disclose the primary events following the photoexcitation of polyynes, fully characterizing the photophysics of these prototypical carbon atomic wires. We find that photoexcitation reaches a high-lying bright state (S_*n*_) and that a very fast IC to a lower-lying dark state (S_1_) takes place with an ∼200 fs time constant. Intraband thermalization processes within the dark state (∼2.5 ps time constant) are also observed. Finally, to complete the description of excited state dynamics, we also measured the decay of the low-lying singlet state (characterized by an ∼500 ps time constant). We found a similar photoexcitation scenario in *sp*-chains with different end groups, such as the monocyano-capped H—(C≡C)_6_—CN and methyl-capped H—(C≡C)_6_—CH_3_ species. Our results, enabled by the combination of the synthesis of stable polyynes, a TA setup with high temporal resolution in the UV, and quantum-chemical modeling of the TA spectra, provide a comprehensive picture of the primary excited state relaxation events in prototypical carbon atomic wires.

## Experimental Section and Methods

### Synthesis

**Figure 1:**
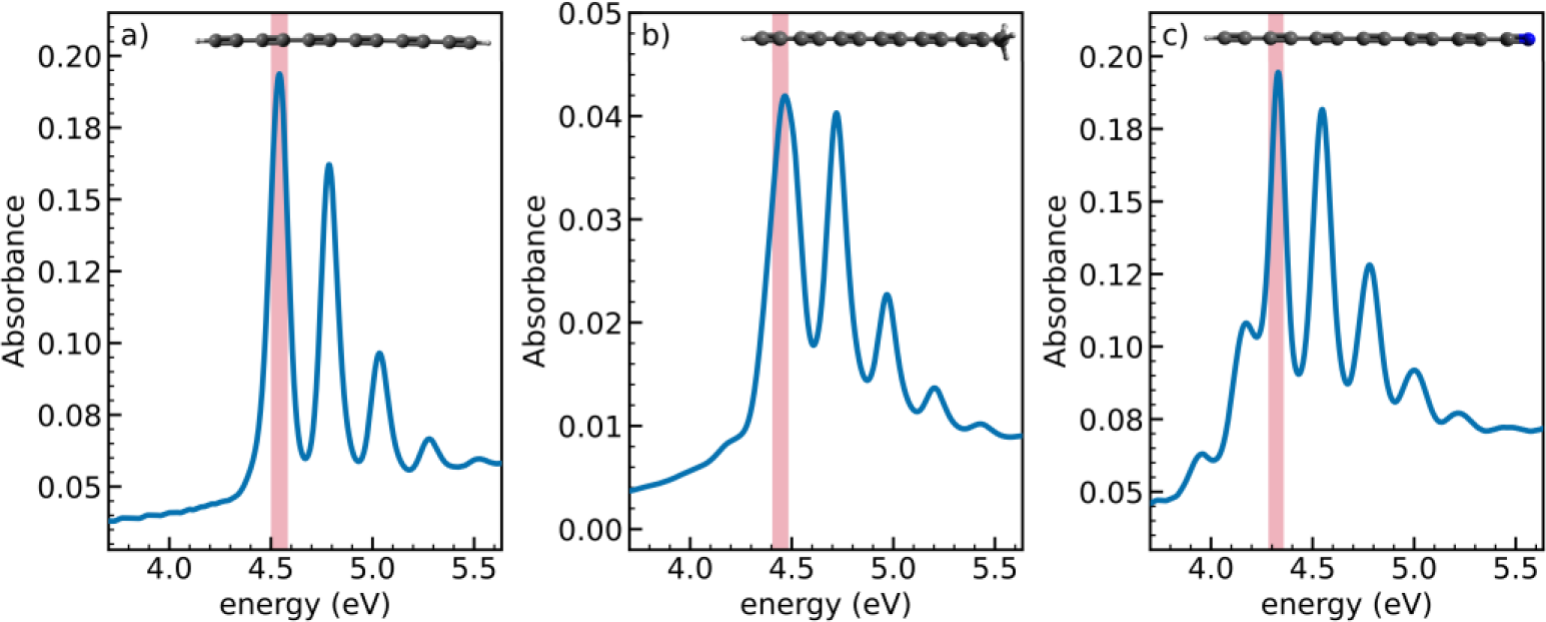
UV–vis absorption spectra and chemical structures of HC_12_—R with different end groups (−R) in water/acetonitrile (10/90 v/v) solutions: (a) −H, (b) −CH_3_, and (c) −CN. Red bands indicate the pump photon energy used in TA experiments.

Based on the method proposed in refs (30) and (31), size- and termination-selected polyynes were collected through reversed-phase high-performance liquid chromatography from a polyyne mixture obtained from the ablation of a graphite target in acetonitrile. Employing this method, we obtained three different water/acetonitrile (10/90 v/v) solutions containing the hydrogen-capped (H—(C≡C)_6_—H), methyl-capped (H–(C≡C)_6_–CH_3_), and cyano-capped H–(C≡C)_6_–CN polyynes, whose concentrations were estimated from UV–vis absorption spectra (optical path: 1 cm) to be 6.61 × 10^–7^ mol/L (see Figure 1a), 6.38 × 10^–8^ mol/L (see Figure 1b), and 1.42 × 10^–7^ mol/L (see Figure 1c), respectively.^32^ For simplicity, we will call H—(C≡C)_6_—H: HC_12_H; H—(C≡C)_6_—CH_3_: HC_12_CH_3_; and H—(C≡C)_6_—CN: HC_12_CN. The samples have been measured immediately after their collection through chromatography. Previous studies ensure their stability in solution for the time required to perform the analysis.^31^

### Computational Methods

The ground state structure of HC_12_H was optimized at the DFT level adopting the range-separated functional ωB97X-D3BJ with the triple-split basis set def2-TZVP. The resolution of identity approximation, namely J for Coulomb integrals and the COSX numerical integration for Hartree–Fock exchange, was applied as implemented in ORCA v. 5.0.3.^33^ Excited state (singlet) calculations were performed at both the TD-DFT and Tamm–Dancoff (TDA) levels of approximation. Ground to excited state vertical transitions (S_0_ → S_*m*_) were computed at the equilibrium ground state geometry, i.e., in the Franck–Condon (FC) region. Excited-to-excited state transitions (S_*n*_ → S_*m*_) were evaluated at the TDA level on top of the ground state (S_0_) geometry. Geometry optimization of the S_1_ state was performed at the TD-DFT level. S_1_ → S_*m*_ transitions were also computed (TDA level) on top of the S_1_ equilibrium geometry. All calculations were performed considering the *D*_∞*h*_ symmetry point group of H—(C≡C)_6_—H. The first triplet state (T_1_) was optimized at the unrestricted (U)DFT level, and the T_1_ → T_*m*_ transitions were computed at both the TD-DFT and TDA levels. Ground and excited state calculations have been also performed by considering a double-hybrid B2PLYP DFT functional^34^ to incorporate the second order Møller–Plesset (MP) corrections to the energies. Data and comparison among functionals (ωB97X-D3BJ vs B2PLYP) and methods (TD-DFT and TDA) are reported in the Supporting Information.

For the cases of methyl-capped (HC_12_CH_3_) and cyano-capped (HC_12_CN) polyynes, the molecular structures of both singlet and triplet ground states (S_0_ and T_1_) were optimized with the ωB97X-D3BJ and def2-TZVP basis sets. Singlet ground to excited state transitions (S_0_ → S_*n*_) as well as singlet and triplet excited state-to-state transitions (namely S_*i*_ → S_*j*_ and T_*i*_ → T_*j*_) were computed at the TD-DFT and TDA levels.

### Ultrafast Transient Absorption Spectroscopy

Ultrafast TA experiments were performed using a home-built setup,^35^ based on an amplified Ti:sapphire laser (Libra, Coherent) generating 100 fs pulses at 800 nm central wavelength (1.55 eV) and 1 kHz repetition rate. A fraction of the laser power was used to pump a broadband visible noncollinear optical parametric amplifier (NOPA). The NOPA output pulses, with a spectrum spanning 500–600 nm (2.07–2.48 eV), were compressed to ∼10 fs duration by chirped dielectric mirrors and successively frequency doubled in a 20 μm thick type I β-barium borate crystal, generating broadband UV pump pulses tunable in the range 250–300 nm (4.13–4.96 eV). The UV pulses were compressed with a MgF_2_ prism pair to nearly transform-limited sub-20 fs duration, as characterized by two-dimensional spectral interferometry.^36^ Broadband probe pulses, covering 320–650 nm (1.91–3.87 eV), were obtained through white light continuum generation by focusing the laser fundamental wavelength in a slowly moving 2 mm thick CaF_2_ plate. The instrumental response function of the system, depending on the probe wavelength, is estimated to be 25–30 fs. The pump energy was adjusted to ≈20 nJ (resulting in a fluence of 80 μJ/cm^2^). For TA measurements on longer time scales, up to 1.3 ns, ∼100 fs pump pulses were used, generated by a narrowband NOPA (∼10 nm bandwidth) tunable in the 2.2–2.3 eV (540–565 nm) range, frequency doubled in a 200 μm thick type I β-barium borate crystal to yield tunable UV pulses. The sample solutions were poured in a 1 mm thick cuvette. After the sample, the transmitted probe was sent to a spectrometer (SP2150 Acton, Princeton Instruments) and detected using a linear image sensor driven by a custom-built electronic board (Stresing Entwicklungsburo GmbH) working at the full laser repetition rate.^37^ For each probe wavelength, the differential absorption (Δ*A*) was measured as a function of the pump–probe delay.

## Results and Discussion

The prototypical polyyne that we study is the hydrogen-capped HC_12_H. The UV–vis absorption spectrum (see Figure 1a) is characterized by a 0–0 band peaking at 4.54 eV (273 nm) followed by well-resolved vibronic replicas, whose position is strongly dependent on the *sp*-carbon chain length and termination.^1,31^ The effect of the terminal groups on the 0–0 transition energy is minimal, and it is reported in Figures 1b and 1c, showing the UV–vis spectra of monomethyl-capped (HC_12_CH_3_) and cyano-capped (HC_12_CN) polyynes. Both groups favor the extension of the π-electron conjugation by lowering the 0–0 transition energy to 4.4 eV (for HC_12_CH_3_) and 4.3 eV (for HC_12_CN). Furthermore, both monocapped species present weak absorption bands at lower energies than the 0–0 transition, namely at 4.2 eV for HC_12_CH_3_ and at 4.0 and 4.2 eV for HC_12_CN. These transitions can be assigned to non-Condon (e.g., Herzberg–Teller) effects, which activate via vibronic coupling low-lying dark electronic excited states.^38−40^

**Figure 2:**
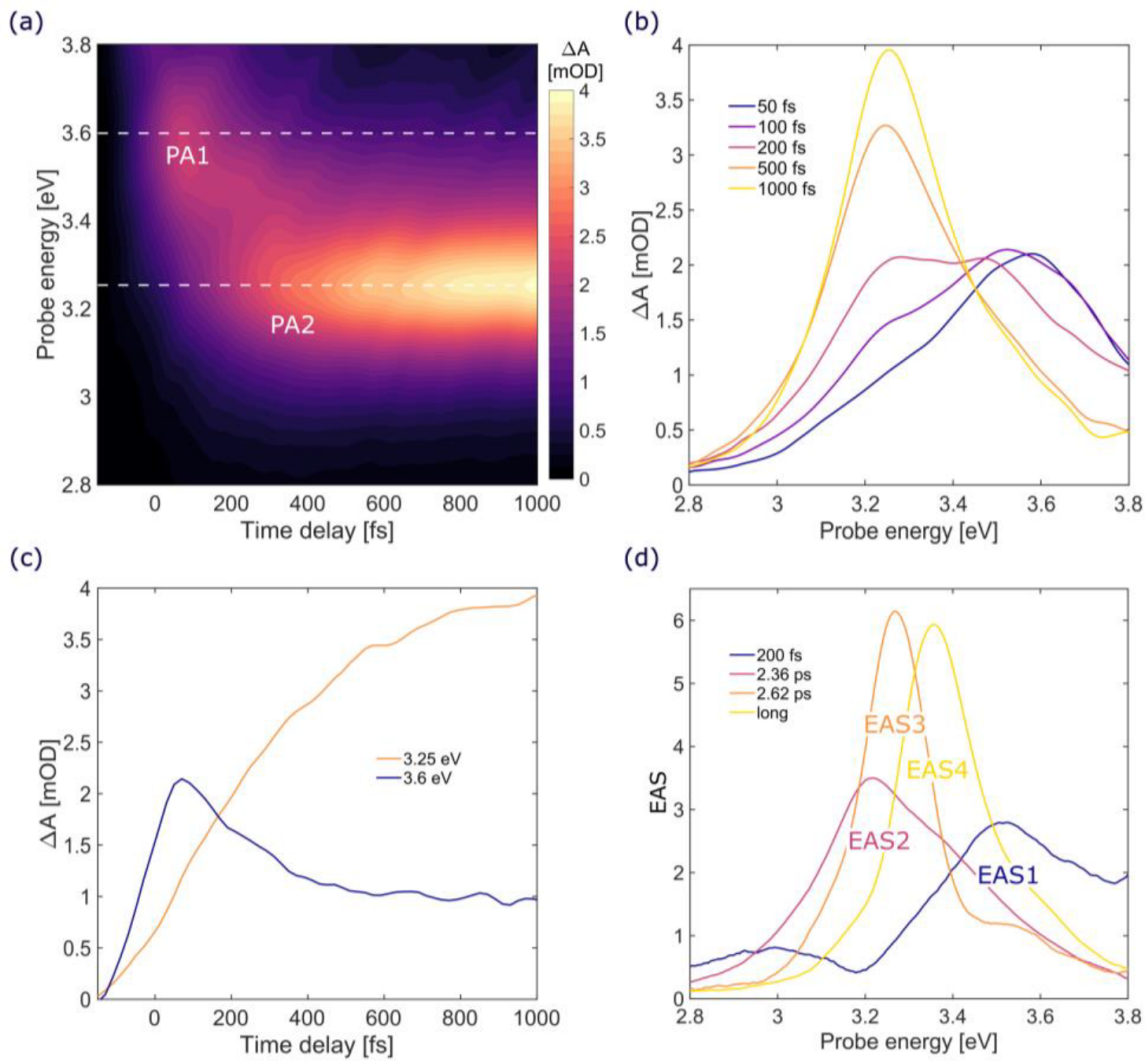
(a–c) TA data of HC_12_H in the first picosecond after excitation (pump at 4.54 eV): (a) TA map as a function of delay and probe photon energy; (b) TA spectra at selected pump–probe delays (from 50 fs to 1 ps); (c) TA dynamics at selected probe photon energies, corresponding to the two PA bands (PA1 blue line and PA2 orange line); (d) EAS obtained from the global analysis of the TA data, with the corresponding time constants.

**Figure 3:**
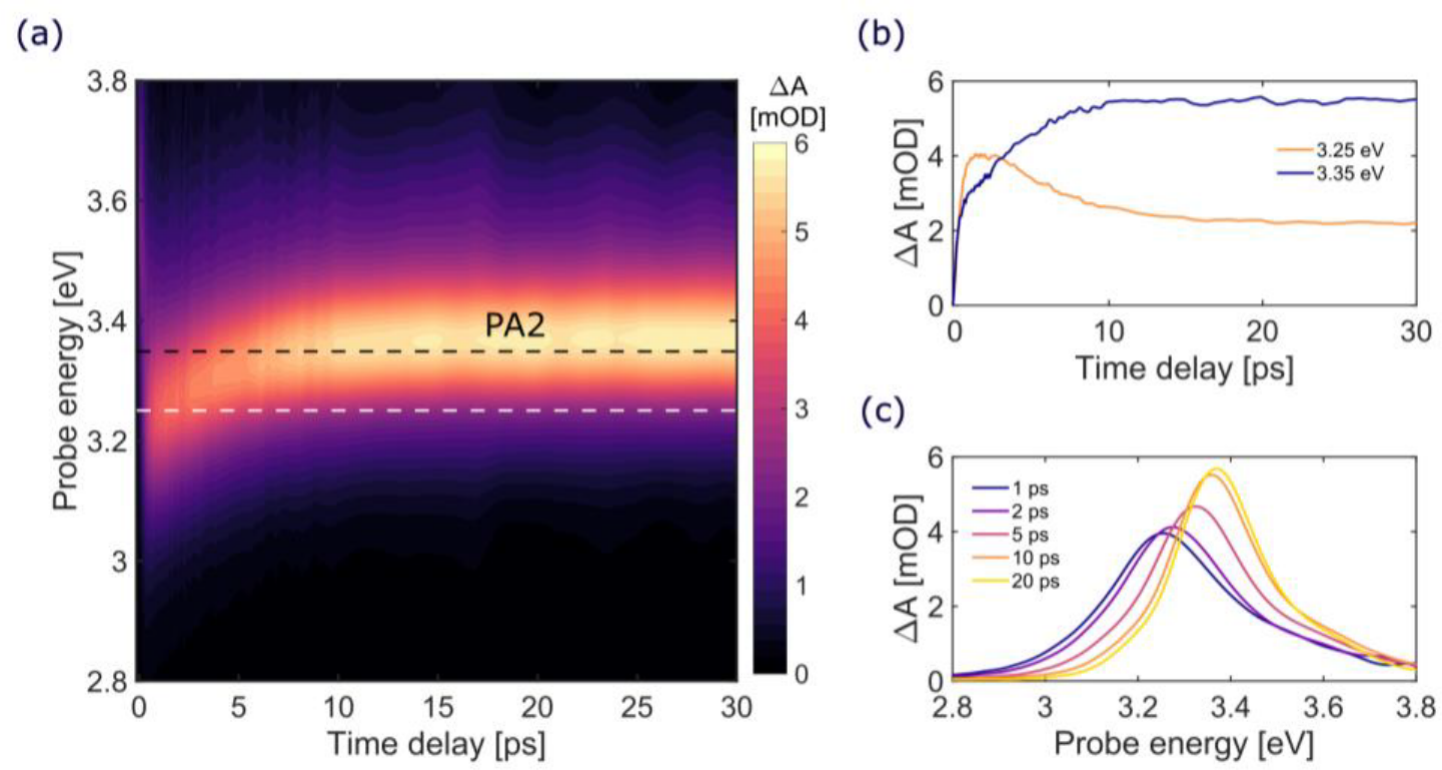
TA data of HC_12_H for time delays up to 30 ps after excitation (pump at 4.54 eV); (a) TA map as a function of delay and probe photon energy; (b) TA dynamics at selected probe photon energies (3.25 eV, orange line; 3.35 eV, dark blue line); (c) TA spectra at selected pump–probe delays (from 1 to 20 ps).

We investigate the early events of ultrafast excited state relaxation of HC_12_H using broadband TA spectroscopy in the UV region with sub-30 fs temporal resolution. We tune the photon energy of the sub-20 fs pump pulses to match the lowest energy absorption peak (at 4.54 eV; see Figure 1a) and probe over a broad spectral region (1.9–3.9 eV). Figure 2a shows a map of Δ*A* as a function of probe photon energy and pump–probe delay up to 1 ps. We observe two positive bands corresponding to photoinduced absorption (PA), the first one centered around 3.60 eV (PA1) and the second one around 3.25 eV (PA2) (see also Figure 2b). The PA1 band rises on the sub-50 fs time scale (blue curve in Figure 2c), which is close to our instrumental response function, and decays on the sub-500 fs time scale. The ultrafast decay of the PA1 band corresponds to the rise of the PA2 band, as confirmed by the presence of an isosbestic point at around 3.4 eV (see Figure 2b). TA data at time delays up to 30 ps are shown in Figure 3. The PA2 band displays a blue-shift by ≈0.2 eV from 3.2 to 3.4 eV on the 10 ps time scale (see Figures 3a and 3c), concomitant with a band narrowing and an increase in oscillator strength (see Figure 3c). Similar data were obtained for the other end-capping groups (Figure S1 in the Supporting Information).

To obtain further information on the excited state dynamics of the system, we performed a global analysis^41^ of TA data using a kinetic model consisting of sequentially interconverting evolution-associated spectra (EAS), with successive monoexponential decays of increasing time constants, which can be regarded as the lifetimes of each EAS. Results of the global analysis are shown in Figures 2d and S2. We observe a first component (EAS1) corresponding to the PA1 band peaking at ≈3.5 eV, which decays with a 200 fs time constant, giving way to EAS2, which corresponds to a “hot” PA2. EAS2 subsequently blue-shifts and narrows, evolving into EAS3 and EAS4 with time constants of 2.36 and 2.62 ps, respectively. We assign these two EAS rising with similar time constants to the nonexponential dynamics which characterizes the blue-shift and the narrowing associated with the thermalization of the PA2 band. EAS4, which peaks at ≈3.38 eV, accounts for the final “cold” form of PA2 and displays a lifetime that is much longer than our experimental observation window.

Overall, the TA data are consistent with the following scenario. Photoexcitation instantaneously (within the time resolution of our TA apparatus) populates the first bright singlet excited state of B_1u_ symmetry (see TD-DFT calculations below), which gives rise to a PA band (PA1 band in Figure 2a peaking at 3.5 eV) to higher-lying singlet states. This PA rapidly decays, giving way to a second red-shifted PA band (PA2 in Figure 2a), which is assigned to a transition from a dark state (S_1_) of A_u_ symmetry to a higher-lying excited state. The matching between the decay of the PA1 band and the growth of the PA2 band is consistent with an IC from the bright (S_*n*_) state to the dark (S_1_) state. In analogy to what was observed with carotenoids, the IC process is ultrafast, characterized by an ∼200 fs time constant and, according to our experimental results, proceeds without the involvement of intermediate excited states. On longer time scales, the spectral narrowing and blue-shift of the PA2 band on the picosecond time scale can be assigned to vibrational relaxation and thermalization of the hot S_1_ state, which induces a blue-shift and a narrowing of its PA signal. This is again in analogy with the blue-shift of the S_1_ PA band observed in carotenoids and attributed to vibrational cooling.^42^

**Figure 4:**
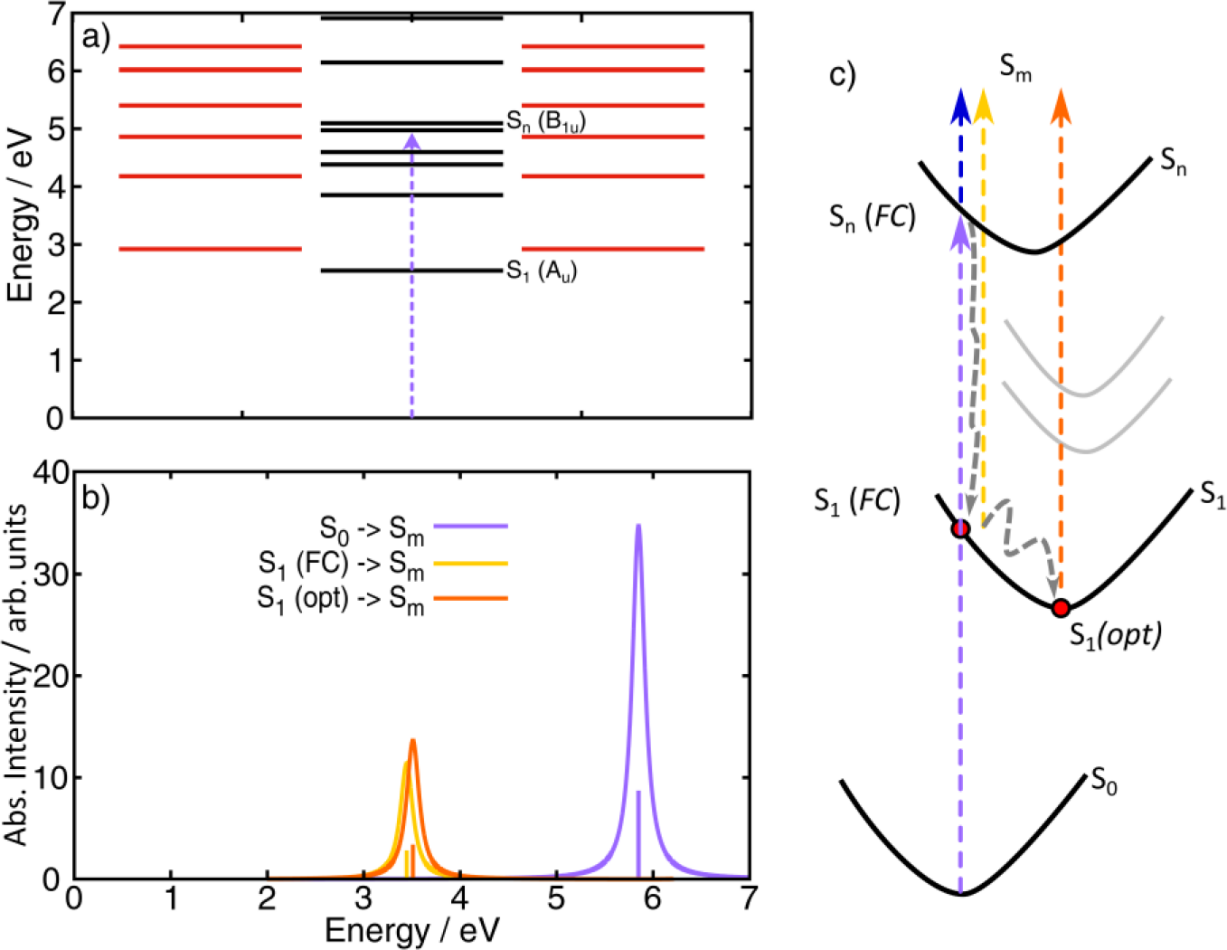
(a) Computed TD-DFT (B2PLYP/def2-TZVP) vertical excited state energies of singlet states; red lines show degenerate dark (i.e., symmetry forbidden) excited states, while black lines refer to not degenerate excited states; S_*n*_ is the dipole-allowed (B_1u_) excited state (violet arrow represents the transition from the ground state), while S_1_ is the first (low-lying) dark (symmetry forbidden, A_u_) state. (b) Computed TDA (ωB97X-D3BJ/def2-TZVP) electronic transitions and spectra for the S_0_ → S_*m*_ transitions (ground to high-lying excited states) (violet line), the S_1_ → S_*m*_ transitions (transient absorption excited-to-excited states) as evaluated at the FC region (i.e., ground state S_0_ geometry) (orange line), and the S_1_ → S_*m*_ transitions as calculated at the relaxed (optimized, opt) S_1_ geometry (dark yellow line); spectra are computed as Lorentzian function centered around the calculated TDA transitions; TDA energies are not scaled. (c) Scheme of the photoinduced relaxation mechanisms involving S_0_, S_*n*_, and S_1_ states; gray arrows sketch the IC from S_*n*_ to S_1_ (FC region) being the first ultrafast process and the cooling mechanisms within the S_1_ state. Violet arrow indicates the ground state photoexcitation, blue arrow indicates the PA1 transition, and dark yellow and orange arrows indicate the hot and relaxed PA2 transitions, respectively. The experimental energy blue-shift of PA2 (Figure 3) is supported by the computed yellow and orange TA spectra (panel b) and sketched by the corresponding arrows (panel c).

We now compare our experimental data with the quantum-chemical calculations on HC_12_H. Excited states were computed using both TD-DFT and TDA methods, considering for each the range-separated functional ωB97X-D3BJ and the double-hybrid B2PLYP. A detailed comparison among methods and functional is reported in the Supporting Information. The use of B2PLYP, through the incorporation of static correlation effects via the perturbative MP2 scheme, lowers the energy of the excited states with respect to ωB97X-D3BJ, leading to values that are in good agreement with the experimental data. For such a reason, Figure 4a reports the excited energy level diagram of HC_12_H, as computed at the TD-DFT level with the B2PYLP functional. The first bright (dipole-allowed) excited state is a high-energy singlet S_*n*_ of B_1u_ symmetry, with an energy of 4.59 eV and an oscillator strength *f* = 5.85. We generally refer here to S_*n*_ as a high-lying singlet excited state: for example, it is S_7_ if computed with TD-DFT and S_10_ if calculated with TDA (see the Supporting Information). S_*n*_ (at both TD-DFT and TDA levels) is characterized by the HOMO → LUMO (π_*y*_–π*_y_) and HOMO–1 → LUMO+1 (π_*x*_–π*_x_) transitions and matches very well the experimental absorption band (Figure 1a) showing the 0–0 vibronic transition centered at 4.54 eV. ωB97X-D3BJ predicts the bright state at a higher energy than B2PLYP, namely at 5.39 eV (*f* = 6.10), however maintaining the HOMO → LUMO (π_*y*_–π*_y_) and HOMO–1 → LUMO+1 (π_*x*_–π*_x_) character. As well-known, ωB97X-D3BJ excited state energies are generally overestimated.^43^ The first excited state S_1_ (A_u_), computed at 2.54 eV at the TD-DFT (B2PLYP) level, is strictly dipole forbidden (*f* = 0.0) because it involves the HOMO → LUMO+1 (π_*y*_–π*_x_) and HOMO–1 → LUMO (π_*x*_–π*_y_) transitions, which are orthogonal to each other and therefore forbidden by symmetry. The same state is computed at 3.16 eV with ωB97X-D3BJ.

From our analysis, the description of the excited state character of HC_12_H in the FC region is independent of the method (TD-DFT vs TDA) and functional (B2PLYP vs ωB97X-D3BJ), all providing very similar results. This point is very relevant for our purposes because it fully justifies the use of TDA (which generally overestimates the excited state energies) for computing the electronic TA spectra using the code ORCA. For this reason, in the following, we discuss the computed TA spectra using the TDA method with the ωB97X-D3BJ functional.

Figure 4b reports the computed S_0_ → S_*m*_ absorption spectrum (violet line) and the TA spectra for two cases, namely (i) the S_1_ → S_*m*_ transition (dark yellow line), as calculated at the FC region (i.e., at the S_0_ equilibrium geometry), and (ii) the S_1_ → S_*m*_ transition (orange line), as computed at the relaxed (optimized) excited state S_1_ geometry. For the S_1_ → S_*m*_ transition at the FC region, an intense absorption band at 3.44 eV is computed. The calculations of the S_1_ → S_*m*_ transition at the S_1_ optimized geometry instead reveal a blue-shift (∼0.07 eV) of the band up to 3.51 eV with an increase of its oscillator strength. The computed S_1_ → S_*m*_ PA band and the predicted blue-shift upon geometry relaxation within the S_1_ potential energy surface well match the observed experimental data (Figure 3c), thus supporting the proposed scenario for the ultrafast IC process.

According to quantum-chemical calculations, HC_12_H shows different equilibrium geometries and structural relaxations depending on the populated electronic state (see Figure S3). While in the ground state (S_0_), HC_12_H shows a clear alternation of single/triple bonds within the *sp*-chain, featuring a bond length alternation parameter BLA (BLA = (⟨*R*_single_⟩ – ⟨*R*_triple_⟩)/*n*, averages of single and triple bonds, with *n* the number of bonds) of the order of 0.16 Å, in the S_1_ state HC_12_H shows a highly equalized (cumulenic-like) structure, resulting in a BLA parameter as low as 0.05 Å in the central part of the *sp*-chain. This is in agreement with previous studies showing a tendency toward equalization upon charge transfer induced by interaction with metal nanoparticles or by the selection of specific end groups.^44−46^ Furthermore, the calculated TD-DFT (ωB97X-D3BJ) energy of the relaxed S_1_ state is 2.88 eV, while the energy of the relaxed T_1_ state is 2.29 eV, which is 0.59 eV below S_1_. The geometry of T_1_ is very similar to that of S_1_, showing a BLA parameter of 0.05 Å in the central part of the *sp*-chain. S_1_ → T_1_ ISC mechanisms might therefore be possible, as already observed for the case of dinaphthyl end-capped polyynes,^23^ given the lower energy of T_1_ with respect to S_1_ and the similarity of the molecular geometries, possibly resulting in effective spin–orbit couplings.

For the methyl- and cyano-capped species (HC_12_CH_3_ and HC_12_CN), the computed TD-DFT energies of the bright S_*n*_ state shift toward lower values, in good agreement with the experimental data (Figures 1b and 1c). The TD-DFT (ωB97X-D3BJ) S_*n*_ vertical energy is 5.39 eV for HC_12_H, 5.33 eV for HC_12_CH_3_, and 5.14 eV for HC_12_CN (unscaled TD-DFT values). Similarly, a red-shift of the triplet states occurs for the cyano-capped species. While for HC_12_CH_3_, the (relaxed) T_1_ energy equals that of HC_12_H (that is, 2.29 eV), for HC_12_CN, the T_1_ minimum red-shifts to 1.88 eV, being the lowest among the three species.

**Figure 5:**
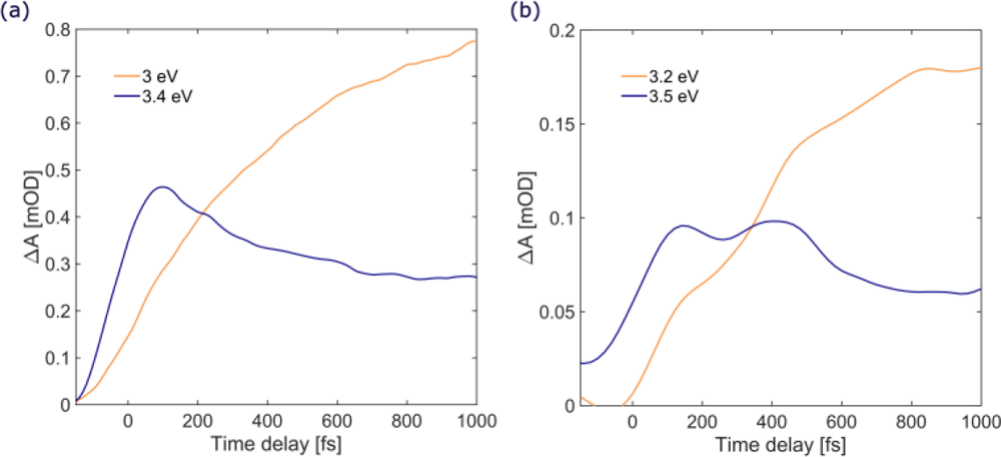
TA dynamics at selected probe photon energies for (a) HC_12_C H_3_ and (b) HC_12_CN.

It is important to note that the observed early excited state relaxation events in polyynes are independent of the termination. TA data measured on HC_12_CH_3_ (Figures 5a and S1a,b) and HC_12_CN (Figures 5b and S1c,d) show similar spectroscopic features, i.e., an IC from the bright S_*n*_ to the dark S_1_ state, with comparable time constants. This experimental observation indicates that end-capped groups do not play a role in the early photoinduced decay events; moreover, this further proves that the simplest possible end-capped *sp*-carbon wires, namely, hydrogen-capped polyynes HC_n_H, are the prototypical systems for 1D carbon-based materials and that the knowledge of their photoinduced decay mechanisms unveils the fundamental photophysics of any polyyne-like species.

**Figure 6:**
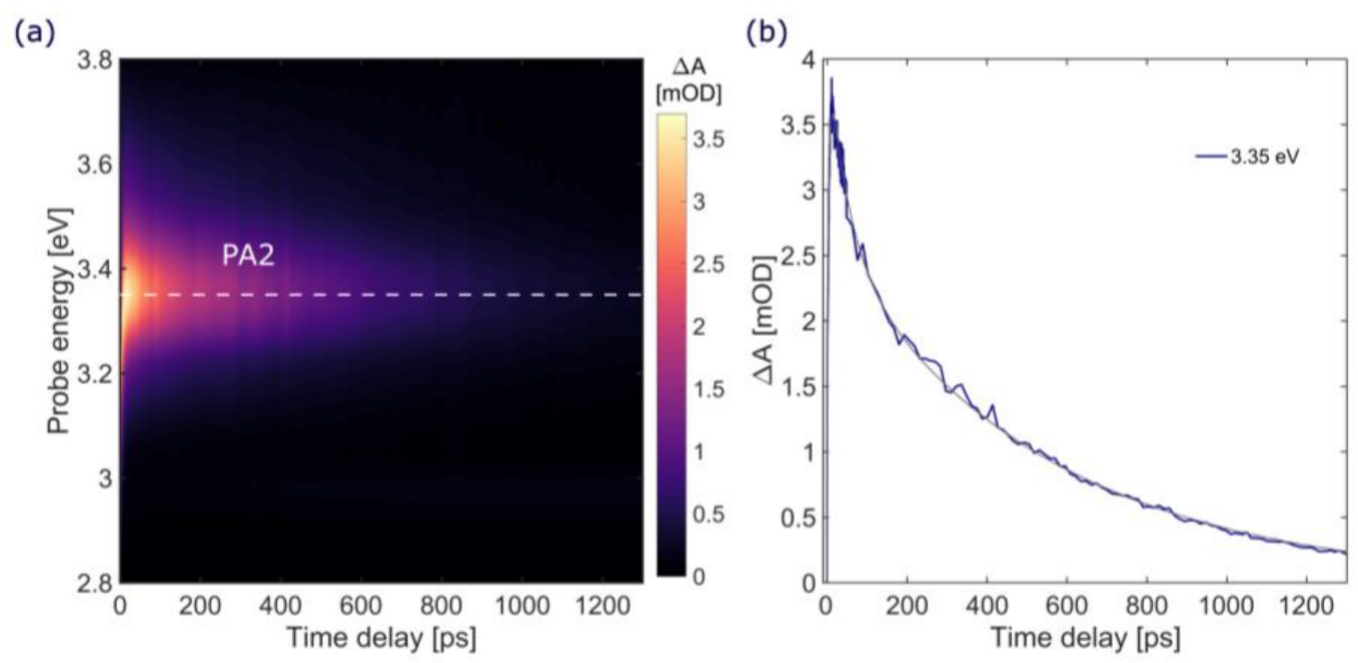
TA data on the long time scale, up to 1.3 ns, for HC_12_C. (a) TA map and (b) TA S_1_ singlet state dynamics at 3.35 eV (blue line) and fitting curve (black line).

Our study focuses on the early excited state relaxation processes in polyynes; nevertheless, for completeness, we also investigated the fate of the S_1_ state. Figure 6a displays the TA map obtained with a narrow-band long (∼100 fs) pump pulse on a long time scale. Here, we can follow the decay of the S_1_ state, which is characterized by two time constants (∼50 and 540 ps, Figure 6b). Similar behavior is observed for all samples (Figure S4). TD-DFT calculations suggest the possibility of an ISC to the triplet state, in particular, for the cyano-capped species, which features a T_1_ energy lower than hydrogen- and methyl-capped chains. We have computed the triplet-to-triplet transitions (T_1_ → T_*n*_) at the TD-UDFT level for each species. For HC_12_CN, the first non-negligible T_1_ → T_*n*_ transition is found at 3.72 eV (T_1_ → T_15_, *f* = 0.15), while for HC_12_H and HC_12_CH_3_ it is predicted at higher energies (∼4.00 eV, *f* = 0.33), out of our experimental probing window. Indeed, for HC_12_CN we could detect the triplet state PA band, corresponding to a T_1_–T_*n*_ excitation peaking at ≈3.77 eV, in very good agreement with our calculations, and observe that its growth (∼500 ps) matches with the decay of the PA of the dark singlet state S_1_ (Figures S1d and S4c,d), as expected and consistently with polyynes featuring other end-capped groups so far reported (120 ps–1.1 ns^12^ and ∼400 ps^24^).

Ultimately, further interesting information would be obtained from the detection of impulsively excited coherent oscillations that could shed light on the vibrational modes coupled to the electronic transitions. However, our temporal resolution enables us to resolve oscillations at frequencies lower than ∼20 THz (∼670 cm^–1^), which are not expected in our systems.^9^

## Conclusions

Despite previous attempts to describe the general photophysics of *sp*-carbon chains, such as polyynes (−C≡C−), and access the early events following photoexcitation, experimental difficulties related to the synthesis of such systems and ultrafast spectroscopy allowed only partial results. Here, by combining the synthesis of prototypical monodispersed hydrogen-capped carbon wires with high temporal resolution ultrafast UV transient absorption spectroscopy and DFT/TD-DFT calculations, we provide a comprehensive description of the primary excited state relaxation processes of polyynes. We prove that the primary photoinduced event is an ultrafast internal conversion from the bright dipole-allowed high-energy singlet state (S_*n*_) to a dark low-energy singlet state (S_1_), and we disclose its ∼200 fs decay time constant. Following internal conversion, a thermalization process in the dark state S_1_ takes place on a few picoseconds time scale. Eventually, the dark singlet state decays in hundreds of picoseconds with an ISC toward a triplet state. Our results provide a comprehensive description of the primary events of excited state relaxations in prototypical carbon atomic wires, where the electronic and steric perturbations, as induced by the end-capping groups, are minimized.

## Supporting Information Available

TA maps, EAS data, DFT optimized geometries, TDA and TDDFT excited state energies (PDF)
