# Supplementary material for: Disclosing Early Excited State Relaxation Events in Prototypical Linear Carbon Chains
Source: J Am Chem Soc. 2023 Aug 1;145(33):18382–90. doi: 10.1021/jacs.3c04163 (PMC10450801; doi:10.1021/jacs.3c04163)
Supplement: Supplementary file 1 — ja3c04163_si_001.pdf [file ja3c04163_si_001.pdf]

Disclosing Early Excited State Relaxation Events in Prototypical Linear Carbon Chains

Piotr Kabaciński,[1] Pietro Marabotti,[2] Daniele Fazzi,[3] Vasilis Petropoulos, [1] Andrea Iudica, [1] Patrick Serafini,[2] Giulio Cerullo,[1,4] Carlo S. Casari, [2]* Margherita Zavelani-Rossi,[2,4]**

[1] Dipartimento di Fisica, Politecnico di Milano, p.za Leonardo da Vinci 32, 20133 Milano, Italy

[2] Dipartimento di Energia, Politecnico di Milano, via G. Ponzio 34/3, 20133 Milano, Italy

[3] Dipartimento di Chimica "Giacomo Ciamician", Università degli studi di Bologna, via F. Selmi 2, 40126 Bologna, Italy

[4] Istituto di Fotonica e Nanotecnologie-CNR, piazza Leonardo da Vinci 32, 20133 Milano, Italy

e-mail address: margherita.zavelani@polimi.it; carlo.casari@polimi.it; daniele.fazzi@unibo.it

SUPPORTING INFORMATION

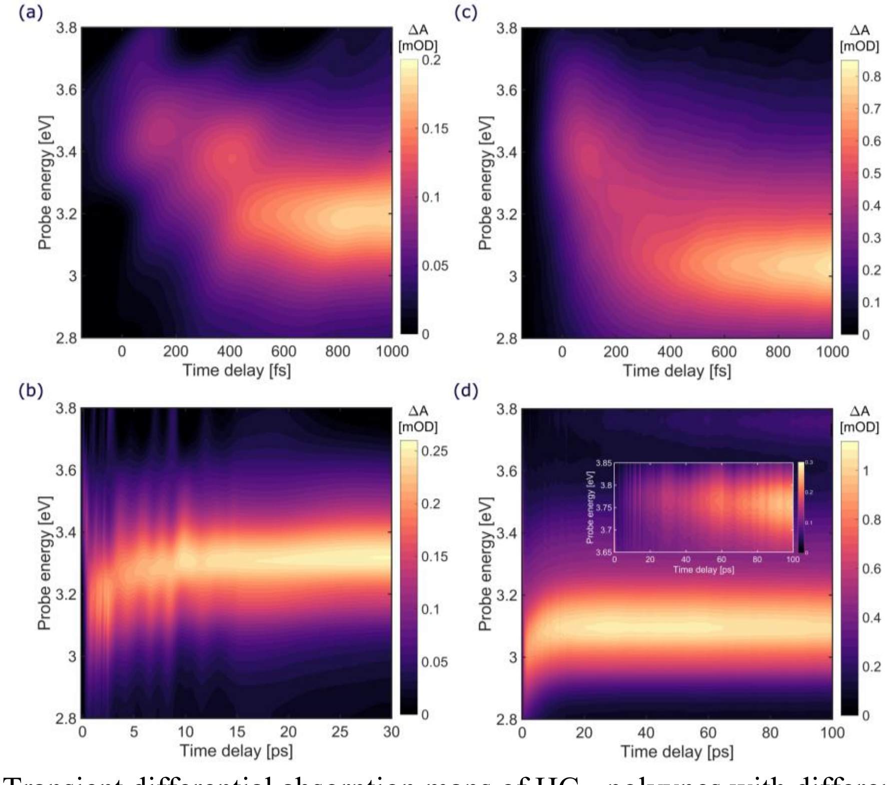

Figure S1. Transient differential absorption maps of HC_{12} polyynes with different end groups (methyl and cyano) on different time scale; (a,b) H−(C≡C)$_6$−CH_3 pumped at 4.44 eV (279 nm); (c,d) H−(C≡C)$_6$−CN pumped at 4.32 ev (287 nm); inset: zoom out of the 3.65-3.85 eV region, rescaled.

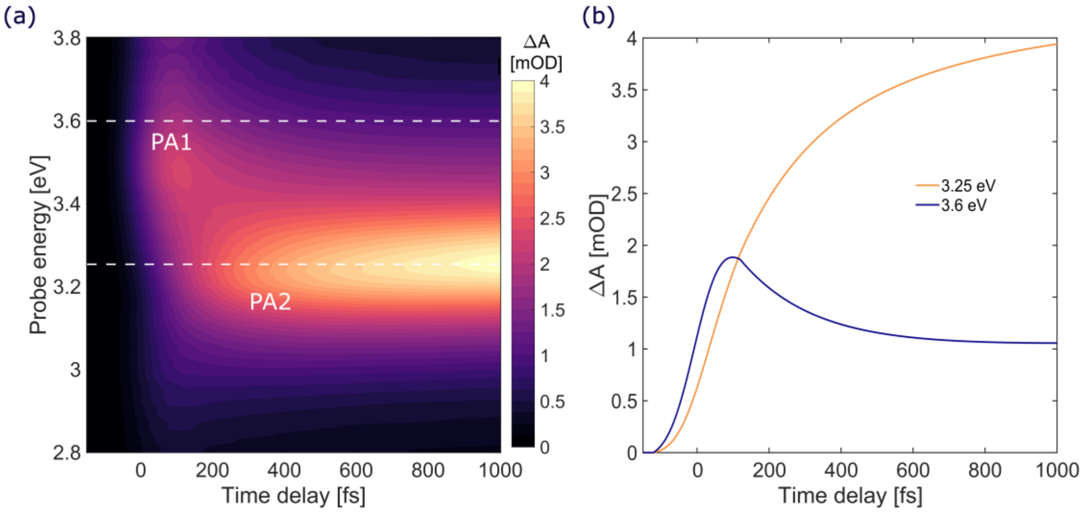

Figure S2. (a) EAS obtained from the global analysis of the TA data of $HC_{12}H$; (b) corresponding TA dynamics at selected probe photon energies, corresponding to the two PA bands (PA 1 blue line and PA 2 orange line)

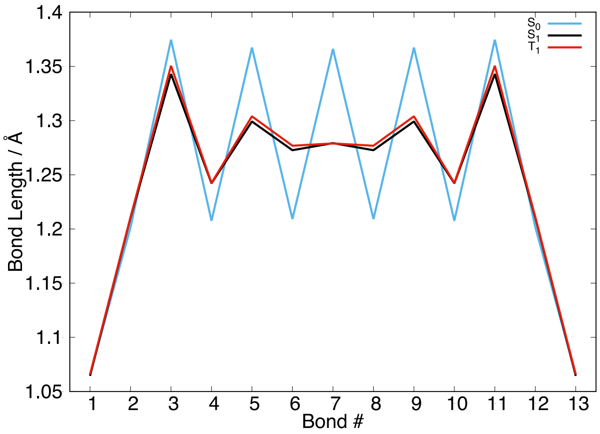

Figure S3: Computed equilibrium bond lengths (Å) for $HC_{12}H$ in the optimized ground state S0 (light blue line), first (dark) excited state S_1 (dark) and triplet state T_1 (red). ωB97X-D3BJ data.

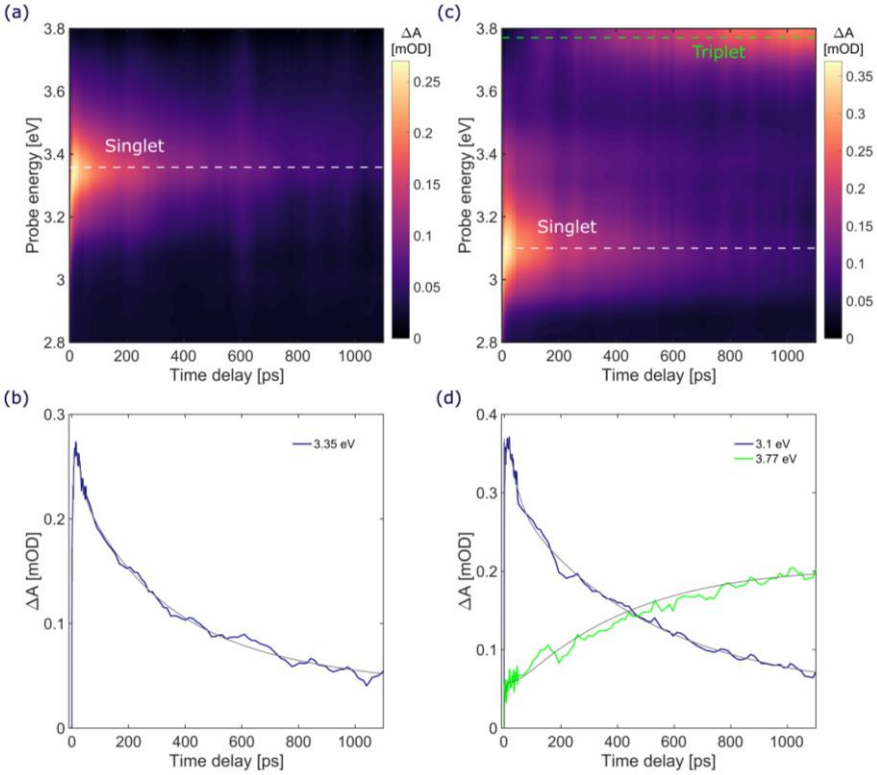

Figure S4. TA data on the long time scale, up to 1.1 ns, (a,c) maps and (b,d) dynamics for selected wavelengths together with bi-exponential fitting curve obtained by global fitting analysis (black lines) for HC_{12} polyynes with different end groups (methyl and cyano); (a,b) $H-(C\equiv C)_6-CH_3$, time constants ~15 ps, ~290 ps; (c,d) $H-(C\equiv C)_6-CN$ time constants ~30 ps, ~500 ps; here the PA of the triplet state (centered at about 3.77 eV) appears.

Computational (DFT, TD-DFT and TDA) data

Optimized geometries

Optimized (wB97X-D3BJ def2-TZVP) ground state (S_0) geometry for $HC_{12}H$

```
14

 H   0.00000008075489     -0.00000042880016      8.10938967974933
 C   0.00000010571831     -0.00000025841304      7.04344348824528
 C   0.00000009266466     -0.00000008622005      5.84190140146244
 C   0.00000006247950      0.00000008566202      4.46727231805684
 C   0.00000002365473      0.00000022482420      3.25970101252309
 C  -0.00000005310736      0.00000031862900      1.89229803243581
 C  -0.00000012981289      0.00000033558428      0.68308119445412
 C  -0.00000019510128      0.00000026930375     -0.68308103852485
 C  -0.00000020240086      0.00000016683383     -1.89229790643410
 C  -0.00000016324765      0.00000003255490     -3.25970095847324
 C  -0.00000007934853     -0.00000007357661     -4.46727235326350
 C   0.00000003811484     -0.00000015484326     -5.84190148128879
 C   0.00000015553554     -0.00000020417574     -7.04344360021106
 H   0.00000026409611     -0.00000022736312     -8.10938978873136
```

Optimized (B2PLYP/D3 def2-TZVP) ground state (S_0) geometry for $HC_{12}H$

```
14

 H  -0.00017207924244      0.00041803745099      8.09680152484801
 C  -0.00011812484375      0.00025037149724      7.03433754015696
 C  -0.00005168089492      0.00007364761763      5.81864303750134
 C   0.00002992449213     -0.00010241542689      4.46722292150165
 C   0.00011768708201     -0.00019886130492      3.23933749583188
 C   0.00013633395143     -0.00028233068579      1.90023072552564
 C   0.00012259981764     -0.00025354994907      0.66805465506586
 C   0.00010489543299     -0.00021831545959     -0.66828260645495
 C   0.00010351774378     -0.00008395681823     -1.90017648682808
 C   0.00004664108122     -0.00007464902566     -3.23925451406323
 C  -0.00004338181496     -0.00015578098689     -4.46714291471396
 C  -0.00009882917516     -0.00006745663819     -5.81857612963456
 C  -0.00009000271994      0.00022886170767     -7.03436453345546
 H  -0.00008750091002      0.00046639802169     -8.09683071528109
```

Optimized (TD-wB97X-D3BJ def2-TZVP) excited state (S_1) geometry for $HC_{12}H$

```
14

 H   0.00000046028401     -0.00000259154718      8.07137675529965
 C   0.00000023869049     -0.00000173959360      7.00685729527599
 C  -0.00000005266064     -0.00000079610272      5.79658881007522
```

C	-0.00000034093049	0.00000020592243	4.45354793473270
C	-0.00000045283280	0.00000107329562	3.21145764919539
C	-0.00000035741116	0.00000177176198	1.91230264736089
C	-0.00000015639080	0.00000215454095	0.63957854488649
C	0.00000005865348	0.00000216687461	-0.63957841609106
C	0.00000020930852	0.00000175893411	-1.91230242275419
C	0.00000027165384	0.00000100607638	-3.21145777021034
C	0.00000020740317	0.00000006278390	-4.45354784518386
C	0.00000005382095	-0.00000095750061	-5.79658895642246
C	-0.00000004481622	-0.00000174597510	-7.00685737764865
H	-0.00000009477235	-0.00000236947077	-8.07137684851577

Optimized (TD-wB97X-D3BJ def2-TZVP) triplet state (T_1) geometry for $HC_{12}H$

14

H	0.00000017481086	0.00000044972401	8.08990840802570
C	0.00000013447646	0.00000024987933	7.02413049709870
C	0.00000008722620	0.00000005312923	5.81312815366413
C	0.00000003216242	-0.00000012014285	4.46266799046614
C	-0.00000001774053	-0.00000022360658	3.22014690508755
C	-0.00000007943104	-0.00000026000466	1.91626229759867
C	-0.00000013577840	-0.00000025907115	0.63943935803695
C	-0.00000020733300	-0.00000024252876	-0.63943930625782
C	-0.00000024576652	-0.00000018523203	-1.91626223818848
C	-0.00000023008352	-0.00000010312039	-3.22014686301095
C	-0.00000010816838	0.00000000337398	-4.46266799611492
C	0.00000004650509	0.00000012304644	-5.81312817665697
C	0.00000020285045	0.00000021563688	-7.02413054535727
H	0.00000034626991	0.00000029891654	-8.08990848439144

Optimized (wB97X-D3BJ def2-TZVP) ground state (S_0) geometry for $HC_{12}CH_3$

17

H	0.01592364241943	-0.03164115369698	8.91516259220102
C	0.01026673357866	-0.02971290646687	7.84931857462199
C	0.00396429470628	-0.02748672859209	6.64768946107539
C	-0.00294962746468	-0.02466360992110	5.27293075292177
C	-0.00855423465083	-0.02175023737810	4.06525012223177
C	-0.01394200084327	-0.01753820639666	2.69778812884552
C	-0.01753537886202	-0.01272433555582	1.48835976635756
C	-0.01970832035771	-0.00547898697795	0.12226446002656
C	-0.01953841002695	0.00295903659551	-1.08738012150632
C	-0.01637197458588	0.01547736341717	-2.45437905408450
C	-0.01047501673671	0.02961143370833	-3.66284899486258
C	0.00063155124838	0.05072651257745	-5.03583727454672
C	0.01077008725596	0.07346070640377	-6.23950651589081

```
  C   0.03479235016600      0.10019958844555     -7.69678589421651
  H   -0.95881550666058      0.32329291870180     -8.09187571520119
  H   0.35487801918986     -0.86711407397764     -8.09046064392988
  H   0.72818292282430      0.86547559242720     -8.05260208855686
```

Optimized (UwB97X-D3BJ def2-TZVP) triplet state (T_1) geometry for $HC_{12}CH_3$

```
17

H       -8.926176   -0.006608    0.009776
C       -7.861359   -0.004812    0.007641
C       -6.649696   -0.003399    0.004996
C       -5.303435   -0.001714    0.001837
C       -4.060505   -0.000046   -0.001107
C       -2.758523    0.000162   -0.003661
C       -1.483877    0.003835   -0.004323
C       -0.205653    0.003328   -0.004322
C        1.070993    0.005205   -0.005224
C        2.368882    0.006040   -0.005983
C        3.615592    0.007338   -0.006550
C        4.956410    0.004930   -0.006557
C        6.172081    0.000537   -0.009427
C        7.623592   -0.001691    0.009709
H        8.016157   -0.693747   -0.738166
H        7.929575   -0.402925    0.982576
H        8.073428    0.984998   -0.116355
```

Optimized (wB97X-D3BJ def2-TZVP) ground state (S_0) geometry for $HC_{12}CN$

```
15

  H      -0.000001     -0.000001      9.433156
  C      -0.000000     -0.000001      8.367003
  C      -0.000000     -0.000000      7.165622
  C       0.000000      0.000000      5.791331
  C       0.000000      0.000000      4.583844
  C       0.000000      0.000001      3.217029
  C       0.000000      0.000001      2.007843
  C       0.000000      0.000001      0.642685
  C       0.000000      0.000001     -0.566711
  C       0.000000      0.000000     -1.931931
  C       0.000000      0.000000     -3.140514
  C       0.000000      0.000000     -4.507927
  C      -0.000000     -0.000000     -5.713015
  C      -0.000000     -0.000001     -7.092296
  N      -0.000001     -0.000001     -8.244652
```

Optimized (UwB97X-D3BJ def2-TZVP) triplet state (T_1) geometry for $HC_{12}CN$

```
15

H       0.000000    0.000000   -9.326275
C       0.000000    0.000000   -8.262042
C       0.000000    0.000000   -7.052671
C       0.000000    0.000000   -5.709223
C       0.000000    0.000000   -4.472288
C       0.000000    0.000000   -3.167922
C       0.000000    0.000000   -1.901532
C       0.000000    0.000000   -0.624852
C       0.000000    0.000000    0.652946
C       0.000000    0.000000    1.935593
C       0.000000    0.000000    3.193262
C       0.000000    0.000000    4.509209
C       0.000000    0.000000    5.736338
C       0.000000    0.000000    7.093003
N       0.000000    0.000000    8.249622
```

Excited States

Excited state energies (vertical transitions as computed on the optimized wB97X-D3BJ geometry) at the TDA level (first 20 excited states). States S1 and S10 are highlighted for clarity.

ABSORPTION SPECTRUM VIA TRANSITION ELECTRIC DIPOLE MOMENTS

State	Energy (cm-1)	Wavelength (nm)	fosc	T2 (au**2)	TX (au)	TY (au)	TZ (au)
**1**	**26514.4**	**377.2**	**0.000000000**	**0.00000**	**0.00000**	**-0.00000**	**0.00000**
2	27136.8	368.5	0.000000000	0.00000	-0.00000	-0.00000	-0.00001
3	27149.1	368.3	0.000000000	0.00000	-0.00000	0.00000	-0.00001
4	34500.2	289.9	0.000000000	0.00000	-0.00000	0.00000	-0.00000
5	35627.7	280.7	0.000000000	0.00000	0.00000	0.00000	0.00001
6	35641.0	280.6	0.000000000	0.00000	-0.00000	0.00000	0.00001
7	43419.3	230.3	0.000000000	0.00000	0.00000	-0.00000	0.00000
8	45057.8	221.9	0.000000000	0.00000	-0.00000	-0.00000	0.00000
9	45073.0	221.9	0.000000001	0.00000	0.00000	-0.00000	-0.00009
**10**	**47171.8**	**212.0**	**8.724275389**	**60.88673**	**-0.00000**	**-0.00000**	**7.80299**
11	51807.5	193.0	0.000000000	0.00000	0.00000	-0.00000	-0.00000
12	53842.9	185.7	0.000000000	0.00000	-0.00000	-0.00000	-0.00001
13	53861.4	185.7	0.000000000	0.00000	0.00000	-0.00000	-0.00001
14	54285.0	184.2	0.000000000	0.00000	0.00000	0.00000	0.00001
15	54289.9	184.2	0.000000000	0.00000	-0.00000	0.00000	-0.00002
16	54311.0	184.1	0.000000000	0.00000	-0.00000	0.00000	0.00000
17	55211.1	181.1	0.000000002	0.00000	0.00000	0.00000	-0.00010
18	58492.8	171.0	0.000000000	0.00000	-0.00000	-0.00000	-0.00000
19	60759.5	164.6	0.000000000	0.00000	0.00000	-0.00000	0.00000
20	60781.6	164.5	0.000000000	0.00000	0.00000	-0.00000	-0.00000

Excited state energies (vertical transitions as computed on the optimized wB97X-D3BJ geometry) at the TD-DFT level (first 20 excited states). States S1 and S10 are highlighted for clarity.

ABSORPTION SPECTRUM VIA TRANSITION ELECTRIC DIPOLE MOMENTS

State	Energy (cm-1)	Wavelength (nm)	fosc	T2 (au**2)	TX (au)	TY (au)	TZ (au)
**1**	**25492.0**	**392.3**	**0.000000000**	**0.00000**	**-0.00000**	**-0.00000**	**0.00000**
2	26373.1	379.2	0.000000000	0.00000	-0.00000	0.00000	-0.00001
3	26390.3	378.9	0.000000000	0.00000	0.00000	0.00000	0.00001
4	33886.8	295.1	0.000000000	0.00000	-0.00000	-0.00000	0.00000
5	35239.8	283.8	0.000000000	0.00000	-0.00000	-0.00000	0.00001
6	35256.1	283.6	0.000000000	0.00000	0.00000	0.00002	-0.00001
7	43066.7	232.2	0.000000000	0.00000	0.00001	-0.00000	0.00000
**8**	**43498.4**	**229.9**	**6.104502598**	**46.20118**	**-0.00000**	**-0.00000**	**-6.79715**
9	44870.7	222.9	0.000000000	0.00000	0.00000	-0.00000	-0.00004
10	44887.8	222.8	0.000000001	0.00000	0.00000	-0.00000	-0.00010
11	51593.0	193.8	0.000000000	0.00000	-0.00000	-0.00000	0.00000
12	53738.3	186.1	0.000000000	0.00000	0.00000	-0.00000	0.00000

13	53758.4	186.0	0.000000000	0.00000	0.00000	-0.00000	0.00000
14	54088.7	184.9	0.000000000	0.00000	0.00000	0.00000	0.00000
15	54094.6	184.9	0.000000000	0.00000	0.00000	-0.00000	0.00002
16	54117.1	184.8	0.000000000	0.00000	-0.00001	0.00000	0.00000
17	54604.9	183.1	0.000000001	0.00000	0.00000	0.00000	-0.00008
18	58348.0	171.4	0.000000000	0.00000	0.00000	0.00000	-0.00000
19	60686.7	164.8	0.000000000	0.00000	-0.00000	-0.00000	0.00000
20	60710.1	164.7	0.000000000	0.00000	0.00000	-0.00000	-0.00000

Excited state energies (vertical transitions as computed on the optimized B2PLYP geometry) at the TDA level (first 20 excited states). States S1 and S10 are highlighted for clarity.

ABSORPTION SPECTRUM VIA TRANSITION ELECTRIC DIPOLE MOMENTS

State	Energy (cm-1)	Wavelength (nm)	fosc	T2 (au**2)	TX (au)	TY (au)	TZ (au)
**1**	**22742.1**	**439.7**	**0.000000000**	**0.00000**	**0.00000**	**0.00000**	**-0.00000**
2	23340.2	428.4	0.000000000	0.00000	0.00000	0.00000	-0.00000
3	23344.2	428.4	0.000000000	0.00000	0.00000	-0.00000	-0.00000
4	32516.8	307.5	0.000000000	0.00000	0.00000	0.00000	0.00000
5	33606.0	297.6	0.000000000	0.00000	-0.00000	0.00000	0.00000
6	33609.2	297.5	0.000000000	0.00000	-0.00000	0.00000	-0.00000
7	42081.1	237.6	0.000000000	0.00000	-0.00000	0.00000	-0.00000
8	43533.3	229.7	0.000000000	0.00000	0.00000	0.00000	0.00000
9	43535.4	229.7	0.000000000	0.00000	-0.00000	-0.00000	0.00000
**10**	**39767.2**	**251.5**	**9.057809931**	**74.98495**	**-0.00000**	**0.00000**	**8.65938**
11	39726.5	251.7	0.000000000	0.00000	-0.00000	0.00000	0.00000
12	39728.1	251.7	0.000000000	0.00000	0.00000	-0.00000	-0.00000
13	40033.4	249.8	0.000000000	0.00000	-0.00000	0.00000	0.00000
14	36068.5	277.3	0.000000000	0.00000	-0.00000	0.00000	-0.00000
15	50270.0	198.9	0.000000000	0.00000	-0.00000	0.00000	0.00000
16	51828.0	192.9	0.000000000	0.00000	-0.00000	0.00000	0.00000
17	51829.2	192.9	0.000000000	0.00000	0.00000	-0.00000	0.00000
18	55945.1	178.7	0.000000000	0.00000	0.00000	-0.00000	0.00000
19	49127.6	203.6	0.000000000	0.00000	0.00000	-0.00000	0.00000
20	48833.2	204.8	0.000000000	0.00000	-0.00000	-0.00000	-0.00001

Excited state energies (vertical transitions as computed on the optimized B2PLYP geometry) at the TD-DFT level (first 20 excited states). States S1 and S10 are highlighted for clarity.

ABSORPTION SPECTRUM VIA TRANSITION ELECTRIC DIPOLE MOMENTS

State	Energy (cm-1)	Wavelength (nm)	fosc	T2 (au**2)	TX (au)	TY (au)	TZ (au)
**1**	**20551.4**	**486.6**	**0.000000000**	**0.00000**	**-0.00000**	**0.00000**	**-0.00000**
2	23544.3	424.7	0.000000000	0.00000	0.00000	0.00000	0.00000
3	23556.6	424.5	0.000000000	0.00000	-0.00000	0.00000	-0.00000
4	31070.7	321.8	0.000000000	0.00000	-0.00000	0.00000	-0.00000
5	33703.0	296.7	0.000000000	0.00000	-0.00000	-0.00000	-0.00000

6	33714.4	296.6	0.000000000	0.00000	0.00000	0.00000	-0.00001
**7**	**37066.8**	**269.8**	**5.850147210**	**51.95854**	**0.00000**	**0.00000**	**-7.20823**
8	41091.7	243.4	0.000000000	0.00000	-0.00000	-0.00000	0.00000
9	43553.7	229.6	0.000000000	0.00000	-0.00000	-0.00000	0.00001
10	43564.1	229.5	0.000000000	0.00000	0.00000	-0.00000	-0.00004
11	39193.2	255.1	0.000000000	0.00000	-0.00000	-0.00000	0.00000
12	39194.8	255.1	0.000000000	0.00000	0.00000	-0.00000	0.00001
13	40117.9	249.3	0.000000000	0.00000	0.00000	-0.00000	-0.00000
14	35334.2	283.0	0.000000001	0.00000	0.00000	0.00000	-0.00007
15	49565.2	201.8	0.000000000	0.00000	0.00000	0.00000	-0.00000
16	51787.4	193.1	0.000000000	0.00000	-0.00000	0.00000	-0.00000
17	51798.4	193.1	0.000000000	0.00000	0.00000	0.00000	0.00000
18	55751.9	179.4	0.000000000	0.00000	-0.00003	0.00001	0.00000
19	48494.3	206.2	0.000000000	0.00000	0.00000	-0.00000	-0.00000
20	48583.1	205.8	0.000000000	0.00000	0.00000	-0.00000	0.00000
